# Influence of different sample preparation strategies on the proteomic identification of stress biomarkers in porcine saliva

**DOI:** 10.1186/s12917-017-1296-9

**Published:** 2017-12-04

**Authors:** Ana Gutiérrez, José Joaquín Cerón, Ebrahim Razzazi-Fazeli, Sarah Schlosser, Fernando Tecles

**Affiliations:** 1 Department of Animal Medicine and Surgery, Regional Campus of International Excellence “Campus Mare Nostrum”, Hospital Veterinario 4 planta, University of Murcia, 30100, Espinardo, Murcia, Spain; 2 VetCore Facility for Research, University of Veterinary Medicine Vienna, Veterinaerplatz 1, A-1210 Vienna, Austria

**Keywords:** Glycoprotein-enrichment, Hexapeptide libraries, Pig, Proteomics, Saliva

## Abstract

**Background:**

The influence of two different sample treatments comprising the enrichment of glycoproteins by boronic acid and dynamic range compression by hexapeptide libraries, on the detection of stress markers in saliva of pigs was evaluated in this study. For this purpose, saliva samples collected before and after the application of an acute stress model consisting of nasal restraining in pigs were processed without any treatment and with the two different treatments mentioned above. Protein separation by two-dimensional gel electrophoresis (2-DE) followed by identification of proteins using MALDI-TOF/TOF mass spectrometry (MS) was used as proteomic technique.

**Results:**

The application of each of the two different sample treatment protocols allowed the identification of unique proteins that could be potential salivary acute stress markers in pigs: lipocalin 1, protein S100-A8 and immunoglobulin M by enrichment of glycoproteins; protein S100-A9, double headed protease inhibitor submandibular gland, and haemoglobin by dynamic range compression; and protein S100-A12 by both protocols. Salivary lipocalin, prolactin inducible protein, light chain of immunoglobulins, adenosine deaminase and carbonic anhydrase VI were identified as potential markers in untreated saliva as well as one of the other treatments.

**Conclusion:**

The use of different procedures allowed the detection of different potential stress markers. Although from a practical point of view, the use of saliva without further treatment as well as the enrichment of glycoproteins are less expensive and easy to do procedures.

## Background

Proteomics studies in saliva are gaining attention both in human and veterinary medicine, as the salivary proteome has the potential for detecting and identified biomarkers of stress and diseases [1].

Stress as an animal welfare alteration leads to considerable financial losses due to an increase in animal susceptibility to disease, a decrease in life expectancy, an impairment of growth and reproduction as well as a decrease in meat quality [2, 3]. During the last 20 years the quantification of cortisol levels in saliva has been used for stress evaluation in porcine. In addition, other markers of stress such as chromogranin A (CgA), immunoglobulin A, testosterone or α-amylase have been measured in saliva [4, 5]. However, there is only one report in which potential new porcine markers of stress are investigated by using proteomic approaches [6].

Dynamic range compression (DRC) by hexapeptide libraries and enrichment of glycoproteins by boronic acid are techniques that have been used for sample enrichment. Both procedures have been applied to human saliva [1, 7]. The dynamic range compression could help to detect potential low-abundance markers which are masked by a subset of abundant proteins that are detected and accurately quantified with traditional sample treatment [8]. On the other hand, enrichment by boronic acid was selected since previous works have confirmed it as a valid non-destructive glycoprotein enrichment methodology for complex samples such as whole saliva in humans [7]. It consists on the covalent reaction between boronic acid and *cis*-diols to form five- or six-membered cyclic esters in alkaline aqueous solution [9] and it is useful for capturing glycosylated molecules from highly complex sample with intact glycan structures [10]. Enrichment of glycoproteins could increase the sensitivity in the detection of carbohydrate-based markers. To the authors` knowledge, there are no previous studies that had explored specifically glycoproteins in saliva as markers of acute stress. However, glycoproteins have been proven to be altered in cultured cells subjected to acute heat stress [11] and disease conditions in vivo such as neoplasms [12].

The objective of this study was to evaluate how the enrichment of glycoproteins using the phenyl boronic acid (PBA) ligand or DRC by hexapeptide libraries could affect the identification of acute stress markers in porcine saliva. For this purpose, these two techniques were applied to aliquots of saliva collected before and after the application of an acute stress model consisting of nasal restraining, and the results were further compared with aliquots of untreated saliva collected the same way but where these techniques were not applied. The saliva samples were analysed by two dimensional gel electrophoresis (2-DE) and differentially altered proteins were identified.

## Methods

### Animals and sampling procedure

Saliva samples of animals subjected to snaring restraint were obtained from 25 pigs of a farm located in the South East of Spain. All animals were males of the commercial crossbred Duroc x (Landrace x Large White) and around 3 to 4 months of age. The pigs were given access ad libitum to water and a diet formulated according to the CP standards recommended by the Spanish Foundation for the Development of Animal Nutrition (Fundación Española para el Desarrollo de la Nutrición Animal 2013. Necesidades nutricionales para ganado porcino. Normas FEDNA. Madrid, Spain. http://www.fundacionfedna.org/sites/default/files/NormasPORCINO_2013rev2.pdf), with 155 g CP/kg and 13.59 MJ of metabolizable energy/kg. They were housed in pens with a minimum space of 0.65 m^2^ per animal (Council of Europe. ETS 123. Strasbourg, France: The Council; 1986. [Last accessed January 5, 2014]. European Convention for the Protection of Vertebrate Animals Used for Experimental and Other Scientific Purposes. http://conventions.coe.int/treaty/en/treaties/html/123.htm) and an average temperature of 23 ± 2 °C.

The pigs were subjected to snaring restraint by using a nose sling according to previous studies [4]. Saliva samples were obtained before (T0) and after 15 min (T15) of the snaring restraint stimuli by allowing the pig to chew sponges for 2–3 min followed by centrifugation of sponge-containing salivette tubes (Sarstedt, Aktiengesellschaft & Co. Nümbrecht, Germany). Two pools of saliva samples of around 50 mL were prepared for T0 and T15 time points, respectively. Each pool was prepared by mixing 2 mL of saliva per animal at the same time point.

### Biochemical stress characterization

For animal stress monitoring CgA and cortisol levels were quantified in saliva samples. An in-housed developed time-resolved immunofluorometric assay was used for CgA quantifications according to the instructions reported earlier [13]. The coefficients of variation were 6.23% and 5.82%, for intra-assay and inter-assay precision, respectively. The limit of quantification was 0.024 μg/mL.

Salivary cortisol measurements were performed using an adaptation of an automated chemiluminescent immunoassay developed for human serum cortisol measurements to porcine saliva [14]. Intra- and inter-assay coefficients of variation were lower than 16%, and the limit of detection was 0.19 ng/mL.

The individual concentrations of CgA and cortisol in saliva samples were log transformed for statistical analysis. Afterwards, an unpaired t test was applied to compare the levels of CgA and cortisol at T0 and T15 using specific statistical software (GraphPad Prism 5, GraphPad Software Inc., La Jolla, United States). The level of significance was set at *P* < 0.05.

### Sample preparation protocols

**Fig. 1:**
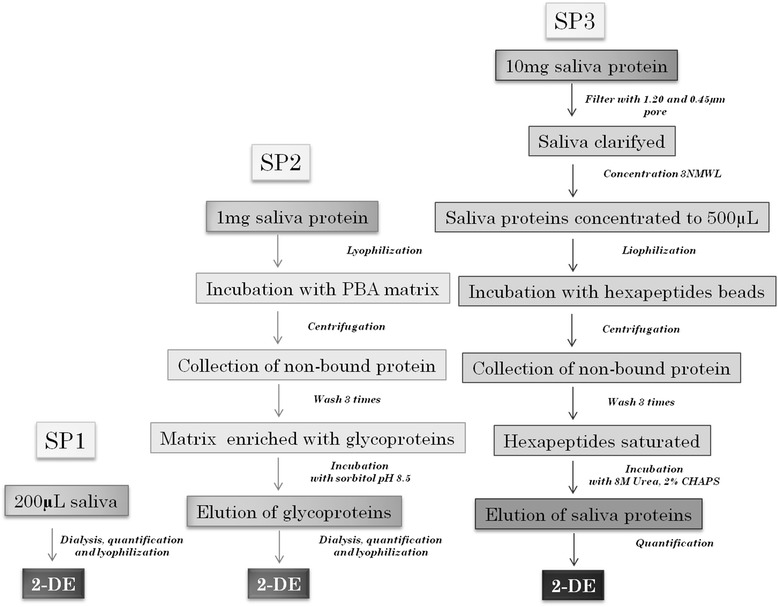
Flowchart of the sample preparation protocols used prior to 2DE-MS analysis for the identification of stress biomarker. SP1: sample preparation 1 (untreated saliva), SP2: sample preparation 2 (glycoprotein enrichment), SP3: sample preparation 3 (dynamic range compression)

The total protein content of the two saliva pools (corresponding to T0 and T15 minutes) was quantified according to Bradford method. Earlier, 3 different sample treatment protocols were performed (Fig. 1). For the sample protocol 1 (SP1), that corresponded to the untreated saliva, three replicates of each salivary pool with 30 μg of total protein were lyophilized and stored at −80 °C.

For the sample protocol 2 (SP2), 1 mg of total protein from each salivary pool was lyophilized and subjected to PBA treatment. A commercial glycoprotein enrichment kit based on PBA matrix was used (NuGel™ Glycoprotein enrichment PBA kit, Biotech Support Group, New Jersey, USA) according to the manufacturer’s instructions. In brief, the freeze-dried PBA saliva prepared of each pool was dissolved in 250 μL of binding buffer, added to 50 mg of PBA matrix and incubated for 10 min. The unbound fraction was discarded after centrifugation for 3 min at 12.750 g. After washing the matrix three times with 350 μL of wash buffer for 5 min, the glycoprotein-enriched fraction was obtained by incubation of the matrix with 300 μL of sorbitol elution buffer for 10 min followed by centrifugation at 12750 x g for 3 min and stored at −80 °C until analysis.

For the third protocol (SP3), 25 mL of saliva from each pool were first filtered using 1.20 μm and 0.45 μm pore size filters, respectively, and then concentrated to 500 µL with centrifugal filter devices of 3 kDa (Amicon Ultra-15, Millipore Corporation, Billerica). Filtration was required since the high level of concentration of the saliva produces the concentration of very small particles of food debris and other saliva components. This protocol was adapted from previous studies [15]. Afterwards, 500 μL of each concentrated pool was lyophilized and subjected to treatment using a commercial kit for DRC (Proteominer Small-Capacity kit, Bio-Rad Laboratories, California, USA) according to the manufacturer’s instructions. In brief, 10 mg of total protein was loaded to beads containing a diverse library of combinatorial peptide ligands. After an incubation of two hours at room temperature in a rotational shaker followed by three washing steps with PBS, proteins were eluted in three consecutive steps through incubation of the beads for 15 min with 20 μL of elution reagent (8 M Urea, 2% CHAPS) followed by centrifugation at 1.000 g for 1 min. The eluate was stored at −80 °C until analysis.

### Two dimensional gel electrophoresis (2DE)

Three replicated gels were performed for each time point in each different sample protocol. For proper comparisons, the same protein amount, 15 μg of total protein was used for all the protocols investigated in this study. The corresponding volume of each sample was subjected to lyophilization in triplicates and the pellets were solubilized in rehydration sample buffer (8 M urea, 2% CHAPS, 2% IPG Buffer, 20 mM DTT). Isoelectric focusing was performed according to the manufacturer’s instructions in 11 cm pH 3–11 NL IPG strips (Immobiline Dry Strip, GE Healthcare Bio-Sciences, Uppsala, Sweden). For the second dimension, IPG strips were first equilibrated for 10 min in 2D–equilibration buffer containing 2% dithiothreitol followed by incubation for 5 min in 2D–equilibration buffer containing 2.5% of iodoacetamide. Electrophoresis was carried out in homemade gradient 10–15% polyacrylamide gels of 140 mm × 140 mm × 1.5 mm at 15 °C and 25 mA/gel. For overall pattern view (analytical gels) and MS analysis (preparative gels), gels were respectively classical and mass compatible silver stained, according to general protocols [16].

2-DE gels were digitalized using an ImageScanner II (GE Healthcare Life Sciences, Uppsala, Sweden) and evaluated by using specific software (Image Master 2D Platinum 7.0, GE Healthcare Life Sciences, Uppsala, Sweden). Gel evaluation consisted of spot detection and matching of protein patterns of three replicated gels of each time point in each different sample protocol. Volumes of single spots were normalized onto the overall spot volume of all spots and thus expressed as % volume. An unpaired t test with Welch’s correction which does not assume equal SDs was used to evaluate spot concentration differences between T0 and T15 in all protocols with specific statistical software (GraphPad Prism 5, GraphPad Software Inc., La Jolla, United States). The level of significance was set at *P* < 0.05.

### Protein identification

Spots that appeared differentially regulated in the different timepoints studied (T0 and T15) were initially identified as compared to identifications of spots obtained in the same positions of the gel in previous studies [17, 18]. In case of no annotation or no correspondence, spots were excised from preparative gels and sent to the VetCore Facility for Research (University of Veterinary Medicine of Vienna, Austria) for protein identification.

2D–spots were washed and destained, followed by reduction with dithiotreitol and subsequent alkylation with iodoacetamide [19]. In-gel digestion with trypsin (Trypsin Gold, Mass Spectrometry Grade, Promega, Madison, WI) was performed as described elsewhere [20]. Peptides were extracted in three consecutive steps with 30 μl of 5% trifluoroacetic acid in 50% aqueous acetonitrile supported by ultrasonication for 10 min, respectively. Subsequently, a vacuum concentrator (Eppendorf, Hamburg, Germany) was used to dry down extracted peptides. In order to concentrate and desalt the dried peptides, C_18_ Zip Tips (microbed) (Millipore, Billerica, MA) were used according to the manufacturer’s instructions.

Peptides (0.5 μl) were mixed 1:1 with matrix consisting of saturated α-cyano-4-hydroxycinnamic acid (PAC target Bruker Daltonics, Bremen, Germany) in aqueous acetonitrile and 0.1% trifluoroacetic acid (30:70, *v*/v), and spotted onto a ground steel target plate. Data were acquired in MS and MS/MS modes on a MALDI-TOF/TOF mass spectrometer (Ultraflex II, Bruker Daltonics, Bremen Germany). Spectra processing and peak annotation were carried out using FlexAnalysis 3.0 and Biotools 3.2 (both Bruker Daltonics, Bremen, Germany).

Processed spectra were searched via Mascot and the software ProteinScape 2.1 (Bruker Daltonics, Bremen Germany) in the UniProt database (taxonomy ‘Sus scrofa’) and the NCBI database (taxonomy ‘all entries’) using the following search parameter: fixed modification carbamido-methylation on cysteine; variable modifications deamidation on asparagine and glutamine, oxidation on methionine as well as formation of pyro-glutamic acid; enzyme specifity trypsin; charge state z = 1, MS tolerance 100 ppm; MS/MS tolerance 1 Da; one missed cleavage allowed. Protein identifications were considered statistically significant where *p* < 0.05 and scores ≥60 as we have reported in previous studies [17].

## Results

### Biochemical stress characterization

**Fig. 2:**
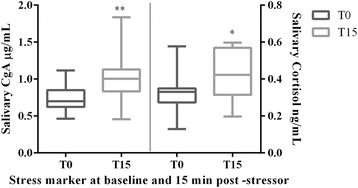
Concentrations of chromogranin A (CgA) and cortisol (μg/mL or ng/mL) in saliva samples of pigs (*n* = 25) before (T0) and after 15 min (T15) of being subjected to a stress model of nose snaring. The plot shows median (line within box), 25th and 75th percentiles (box), 5th and 95th percentiles (whiskers) and outliers (°). Asterisk represents the statistically significant differences from each group. *level of significance *P* < 0.05. **level of significance *P* < 0.01

The results of the acute stress markers quantified in saliva samples of pigs subjected to the experimental stress stimuli appear in Fig. 2. A statistically significant increase was observed after 15 min of the stressor application in comparison to the basal level for both biomarkers, CgA (1.43-fold, *p* = 0.0077), and cortisol (1.30-fold, *p* = 0.0342). Results obtained for the pooled saliva samples at T0 vs. T15 were 0.72 vs. 1.04 μg/mL for CgA, and 0.37 vs. 0.51 ng/mL for cortisol.

### Sample preparation protocols comparison

The amount of original pooled saliva sample needed to perform the complete analysis was completely different between protocols. For SP1, which corresponds to untreated saliva, a volume of 54 μL of each pooled saliva sample was used. In contrast, 1.2 mL of each pooled saliva was needed for SP2 and 25 mL for SP3 protocols. The mean % of the total amount of protein recovery was 5.9% and 1.2% for SP2 and SP3, respectively.

The time spent to perform each protocol was similar, oscillating between 13 and 19 h. However, the labour invested was different since sample preparation in SP1 required 12 h of dialysis and 3 h of lyophilization (total 15 h). In contrast, SP2 needed two lyophilization steps of 3 h, 12 h of dialysis and 50 min for the enrichment of glycoproteins - a multistep procedure with several incubations and centrifugations (total 19 h). To perform SP3, a filtration step was needed, followed by 5 h of saliva concentration, a lyophilization step of 5 h, and 3 h for the DRC of proteins, a multistep procedure with several incubations and centrifugations (total 13 h).

### Proteomic analysis comparison

**Fig. 3:**
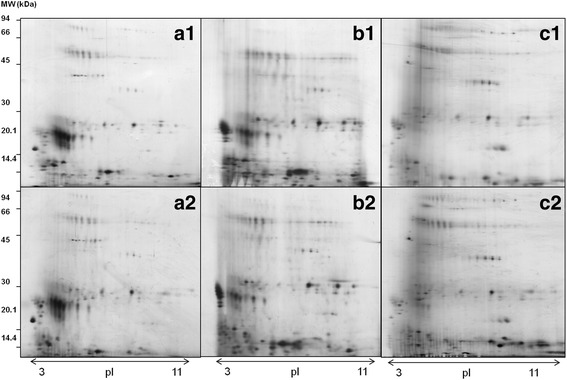
Representative images of the salivary protein patterns obtained by 2-DE after three different sample preparation approaches, SP1 (untreated saliva, **a**), SP2 (glycoprotein enrichment, **b**), SP3 (dynamic range compression, **c**) on saliva samples from animals at T0 (1) and T15 (2) after being subjected to a stress model of nose snaring

Figure 3 shows gel images obtained with SP1 (a), SP2 (b) and SP3 (c) protocols in basal (1) versus stress (2) conditions. Untreated saliva contained the most abundant proteins within this fluid (Figure 3a_1_). SP2 depleted non-glycosylated proteins which resulted in the disappearance of several 2D–spots and the appearance of some new 2D–spots in the more acidic pH range that were previously undetectable in untreated saliva (Figure 3b_1_). SP3 showed also new spots that were not detectable in untreated saliva (Figure 3c_1_). The comparison between the gel images obtained in basal versus stress (Figure 3a_2_, b_2_ and c_2_) conditions showed similar number of matched 2D–spots in the case of SP1 and SP2 (83 and 84, respectively). However, only 23 matched 2D–spots were observed when SP3 was used.

**Fig. 4:**
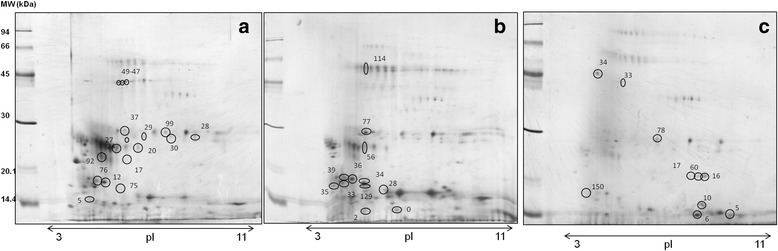
Representative images of the 2D–spots successfully identified (circles) by MALDI-TOF/TOF MS or western blot from salivary protein patterns obtained in SP1 (**a**) SP2 (**b**) and SP3 (**c**). Numbering corresponds with data in Tables 1 and 2

The three protocols allowed the identification of several proteins that could be considered as potential markers of acute stress in porcine saliva (Fig. 4, Tables 1 and 2). By applying the SP1 protocol, various proteins differentially regulated in stress vs. basal conditions were successfully identified. These proteins were salivary lipocalin (5 spots), light chain of immunoglobulins (4 spots), prolactin inducible protein (3 spots), adenosine deaminase (3 spots) and carbonic anhydrase VI (1 spot). The application of SP2 protocol revealed prolactin inducible protein (1 spot), salivary lipocalin (1 spot) and carbonic anhydrase (1 spot) as differentially regulated proteins in compliance with the SP1 protocol, as well as four proteins not detected with other protocols: lipocalin 1 (1 spot), immunoglobulin M (1 spot) and protein S100-A12 and A8 (1 spot each). Finally, in SP3 adenosine deaminase (1 spot) and light chain of immunoglobulins (1 spot) were obtained to be differentially regulated in stress vs. basal as in SP1, and protein S100-A12 as in protocol SP2. In addition, performing SP3 enabled the identification of three proteins that were not differentially regulated in the other two protocols, specifically protein S100-A9 (2 spots), double headed protease inhibitor submandibulary gland (1 spot) and haemoglobin (1 spot).

**Table 1:** Identification of 2D–spots which appeared differentially regulated under stress (T15) in comparison to basal levels (T0) from pooled saliva samples of pigs subjected to snaring restraint

Sample preparation	Spot number	Mean T0	Mean T15	*P* value	Protein identification^a^
SP1	5	0.23	1.05	0.0001	Prolactin inducible protein
	20	0.53	1.07	0.0011	Salivary lipocalin^c^
	76	1.68	0.99	0.0011	Prolactin inducible protein^c^
	49	0.39	0.28	0.0050	Adenosine deaminase
	48	0.29	0.21	0.0064	Adenosine deaminase
	30	0.13	0.20	0.0089	Light chain of immunoglobulins^b^
	29	0.14	0.44	0.0148	Light chain of immunoglobulins^b^
	75	0.04	0.61	0.016	No identification
	24	2.85	1.45	0.0218	Salivary lipocalin
	47	0.24	0.15	0.0219	Adenosine deaminase
	17	0.18	0.52	0.0243	Salivary lipocalin
	99	0.22	0.15	0.0295	Light chain of immunoglobulins^b^
	27	0.20	0.50	0.0353	Salivary lipocalin
	37	0.31	0.58	0.0385	Carbonic anhydrase VI
	92	2.97	1.30	0.0405	Salivary lipocalin
	12	1.62	1.02	0.0421	Prolactin inducible protein^c^
	28	0.14	0.26	0.0481	Light chain of immunoglobulins^b^
SP2	34	0.21	0.86	0.0006	No identification
	129	0.13	0.13	0.0007	No identification
	36	1.80	1.15	0.0032	Prolactin inducible protein^c^
	28	0.25	0.16	0.0046	No identification
	0	0.63	0.42	0.0053	Protein S100-A12
	77	0.53	0.83	0.0068	Carbonic anhydrase VI
	35	2.59	1.9	0.0104	Lipocalin 1
	38	1.10	0.45	0.0139	Prolactin inducible protein
	56	1.42	0.86	0.0161	Salivary lipocalin
	2	0.42	0.64	0.0379	Protein S100-A8
	114	0.31	0.18	0.0410	Immunoglobulin M
	33	1.40	0.62	0.043	No identification
SP3	10	0.77	1.79	0.0002	Double headed protease inhibitor submandibular gland
	6	1.76	3.55	0.0006	Protein S100-A12
	136	0.09	0	0.0020	No identification
	60	1.13	0.68	0.0092	Protein S100-A9
	5	1.10	2.00	0.0101	Hemoglobin^c^
	16	2.10	1.23	0.0104	Protein S100-A9
	33	0.33	0.15	0.0170	Adenosine deaminase
	78	0.85	0.62	0.0260	Light chain of immunoglobulins^b^
	150	0.90	0.25	0.0318	No identification
	34	0.34	0.71	0.0326	No identification
	17	0.59	0.36	0.0463	No identification

SP1: sample preparation 1, untreated pooled saliva. SP2: sample preparation 2, glycoprotein enrichment fraction of pooled saliva samples. SP3: sample preparation 3, dynamic range compression of pooled saliva samples

^a^Identifications obtained by comparison to previous MS data [16, 17]. ^b^Identifications obtained by comparison to previous Western blot data [17]. ^c^ MS identifications performed in the present study (Table 2)

**Table 2:** Protein identification of 2D–spots shown in Fig. 4 by MALDI-TOF/TOF mass spectrometry

Spot ID	Acc No.	Protein identified*	MW	pI	Mascot Score	N	Cov%	m/z	Range	Peptide Score	Peptide Sequence
20	P81608	Salivary lipocalin	21.6	5	107.1	2	8.9	899.5607 1291.6910	59–65 132–141	35.2 71.9	R.VFVEHIR.V K.TFQLMEFYGR.K
76	gi|545882006	Prolactin-inducible protein	16.8	5.1	311.8	4	27.3	1318.7711 1449.6481 1947.1172 1964.1477	111–122 89–99 123–140 123–140	47.8 49.6 97.3 117	K.SMVITATVDIIR.Q K.YTSCLCDDYPR.K R.QLGICPQDQAVIPIAANR.F R.QLGICPQDQAVIPIAANR.F
12	gi|545882006	Prolactin-inducible protein	16.8	5.1	355.9	4	27.3	1318.7716 1449.6317 1947.0736 1964.1255	111–122 89–99 123–140 123–140	66.8 74.5 91.8 122.9	K.SMVITATVDIIR.Q K.YTSCLCDDYPR.K R.QLGICPQDQAVIPIAANR.F R.QLGICPQDQAVIPIAANR.F
26	gi|545882006	Prolactin-inducible protein	16.8	5.1	395.8	4	27.3	1318.7446 1449.6003 1947.0135 1964.1257	111–122 89–99 123–140 123–140	63 75.2 114.1 143.6	K.SMVITATVDIIR.Q K.YTSCLCDDYPR.K R.QLGICPQDQAVIPIAANR.F R.QLGICPQDQAVIPIAANR.F
5	P01965	Hemoglobin subunit alpha	15	9.6	145.9	2	22	1422.6998 1876.8757	17–31 41–56	64.3 81.5	K.VFGGQAGAHGAEALER.M K.TYFPHFNLSHGSDQVK.A

Acc. No. = accession number. MW = molecular weight in kDa. pI = isoelectric point. N = number of peptides. Cov = sequence coverage. * Statistically significant identification *P* < 0.05. Theoretical pI and Mw values were sourced from the ExPASy Proteomics Server (Swiss Institute of Bioinformatics, Geneva)

Data about the regulation of the proteins that were found differently expressed before and after the stress application appear in Table 3. The proteins that are up-regulated after stress were light chain of immunoglobulins, carbonic anhydrase VI, protein S100-A12, protein S100-A8, double headed protease inhibitor submandibulary gland and haemoglobin. On the other hand, the proteins that appeared down-regulated after stress in comparison to basal conditions were prolactin inducible protein, salivary lipocalin, adenosine deaminase, protein S100-A9, lipocalin 1 and immunoglobulin M.

**Table 3:** Differential expression of proteins successfully identified by MS analysis under stress conditions in porcine saliva samples subjected to different sample preparation protocols

Protein name	Mean Basal (T0)	Mean Stress (T15)	Regulation	Number of spots identified
Salivary lipocalin	1.36	0.95	Down	6
Prolactin inducible protein	1.29	0.94	Down	5
Light chain Igs	0.30	0.34	Up	5
Adenosine deaminase	0.31	0.20	Down	4
Carbonic anhydrase VI	0.43	0.71	Up	2
Protein S100-A12	1.20	1.99	Up	2
Protein S100-A9	1.08	0.97	Down	2
Lipocalin 1	2.60	1.97	Down	1
Protein S100-A8	0.43	0.65	Up	1
Ig M	0.31	0.18	Down	1
Double headed protease inhibitor submandibular gland	0.77	1.79	Up	1
Haemoglobin	1.10	2.00	Up	1

## Discussion

Saliva presents a large protein complexity with proteins from several different origins such as salivary glands secretions, plasma filtrates, cellular and bacterial derivatives, bronchial and gastrointestinal fluids and others. Moreover, many informative analytes are generally present in low amounts and the protein profile of saliva is dominated by subset of abundant proteins. Thus to improve detection and visibility of proteins expressed at lower levels in saliva, several depletion strategies has been reported such as enzyme-substrate depletion, immune-depletion or combinatorial peptide ligand library in humans [8]. Moreover, it has been reported that disease specific glycoprofile changes of a specific protein or protein subset are considered to better reflect given physiologic condition compared to an overall dys-regulation of a particular protein [21]. However, the specific enrichment and purification of glycoproteins/glycopeptides is essential prior to mass spectrometric analysis since some glycoproteins are found in low abundance [22]. Taken into account all the statements detailed above, the use of techniques for dynamic range compression or glycoprotein profile of saliva seems to be of value for increasing the discovery of salivary markers and has been applied in the present study in pigs for stress biomarker identification and compared to untreated saliva. A homogeneous population of animals with similar sex, age and weight was used in order to minimize interindividual variability.

Pooled salivary samples from animals subjected to restrain stress at different time points, basal (T0) and stress peak (T15), were used in this study. The increased concentrations of CgA and cortisol at T15 indicated that the experimental model used was suitable to produce stress, as it has been previously described [13].

The main differences between the sample treatment protocols used in the present study were the amount of initial sample (54 μl in SP1 vs. 25 mL in SP3) and the labour (SP1 being less complex than SP3) required for completing the treatments. In our study, a protocol for glycoprotein enrichment was applied using a commercial PBA resin as described for human saliva [7]. Moreover, the use of DRC by hexapeptide libraries has been successfully applied to human saliva using the same reagents as in our study [1].

The application of three different protocols for processing samples enabled the detection of a total of 12 proteins that showed significant changes in expression before and after stress. However, only three proteins were observed in SP1 and SP2 simultaneously (prolactin inducible protein, salivary lipocalin and carbonic anhydrase VI), two in SP1 and SP3 (adenosine deaminase and light chain of immunoglobulins) and one in SP2 and SP3 (protein S100-A12). The remaining 6 proteins suggested as markers of stress were reported according to the results of only one protocol (lipocalin 1, protein S100-A8 and immunoglobulin M in SP2; and protein S100-A9, double headed protease inhibitor and haemoglobin in SP3). The reason why none of the markers proposed in the study appeared in all three protocols simultaneously is very likely the targeted nature of the applied SP2 and SP3 protocols as in SP2 the main proteins which were detected are the glycosylated ones whereas in SP3 low abundant proteins predominate. It is important to note that even when using the same samples, not only different sample treatment protocols but also different mass spectrometry platforms produced completely different biomarker profiles, and therefore more than one analytical proteomic platform can be employed for wide the range of biomarkers detection [23].

All proteins identified in the present study could be considered as novel potential markers of acute stress in pigs, although none of these proteins were identified in a previous investigation in which changes of the saliva proteome of pigs were studied under different acute stress stimuli [6]. These differences could be explained by the application of different proteomic approaches, including sample preparation and staining of 2D–gels [16] as well as by the different stressor applied to the animals [24]. In contrast, some of them (salivary lipocalin, lipocalin 1, double headed protease inhibitor protein, adenosine deaminase, proteins S100-A8, S100-A9 and S100-A12) were previously described as potential markers of porcine systemic disease using conventional proteomic approaches [17]. Lipocalins are involved in immune response modulation and regulation of cell homeostasis [25]. S100A8, S100A9, and S100A12 are predominantly expressed in phagocytes and are strongly associated with pro-inflammatory functions, inducing chemotaxis, neutrophil adhesion and secretion of pro-inflammatory mediators [26, 27]. All these proteins, as well as double headed protease inhibitor, are overexpressed in saliva of pigs with systemic disease [17], but to the authors’ knowledge little information is available about the function of this proteins under stress conditions.

Adenosine deaminase is an enzyme considered as a biomarker of cell mediated immunity [28] since increases in serum due to cell damage [29] or increase in the number of T lymphocytes [30]. It is also present in saliva and increases in oral diseases such as squamous cell carcinoma of the tongue [31]. In pigs, salivary adenosine deaminase is up-regulated due to systemic inflammation [17]. Carbonic anhydrase VI is the only isozyme of the carbonic anhydrase family that is secreted in saliva [32]. It participated in several functions such as ion transport, secretory processes and saliva production, regulation of the pH and buffering capacity of the saliva [33], olfaction and taste [34, 35]. A relationship between its secretion and the sympathetic nervous system has been reported [34]; although no increase was detected in pigs after acute stress [36] but in pigs with systemic disease [17].

Prolactin inducible protein in saliva is involved in immune regulation in the mouth, having a role in tumor progression, preventing bacterial adhesion and infection [37]. An increase of this protein in saliva has been reported in humans after acute stressor [38]. Salivary IgM derives from crevicular fluid or from plasma leakage [39]. An increase on IgM titre, as well as IgA and IgG, has been reported due to psychological stress in students [40]; so it could be a potential stress marker. Light chains of immunoglobulin interact with neutrophils and mast cells, stimulating chronic inflammation via activation of specific immune cells [41]. No reports have been found light chains of immunoglobulins in saliva and stress, although a decrease has been reported after physical exercise in humans [42].

### Limitations

This study has some limitations that need to be discussed. Ideally reproducibility studies of the protocols should have been performed. This is especially important in SP2 and SP3 where a pretreatment of the sample were performed and the lack of replication might bias the results somewhat. Budget limitations and the high amount of saliva required for the SP3 procedure did not allow us to do these studies. Overall without these reproducibility studies the results should be taken with caution.

Another limitation related to SP2 protocol is that a specific staining for glycoproteins was not performed to ensure the usefulness of the PBA for enriching glycoproteins, since we did not have enough sample volume left after performing the analytical protocols. However several reports have demonstrared that boronic acid is adequate for glycoproteins enrichment prior mass analysis [10, 43]. In addition regarding SP3, this protocol produces a loss in the protein detected, since very high abundance proteins quickly saturate their hexapeptide baits, such that a significant proportion of these proteins does not bind (i.e., is depleted) and removed in the flow-through [1]. Although expected, the high amount of lost protein could be considered as a limitation of this protocol.

It is also important to point out the possible blood contamination of saliva samples, since hemoglobin was detected, although none of the saliva samples used in the present study presented a hemolytic color. In addition, the use of liquid chromatography tandem mass spectrometry (LC-MS/MS) instead of MALDI could have increased the sensitivity of the protein identification, which can be considered another limitation of this study.

## Conclusions

In conclusion, the use of different procedures allowed the detection of different potential stress markers..Untreated saliva as well as the application of a glycoprotein enrichment protocol required only a low amount of saliva sample and provided promising results with modest labour. On the contrary, the use of DRC by hexapeptide libraries prior to 2DE-MS analysis would not be recommended due to the high amount of saliva needed, the complexity of the sample preparation and the large source of potential variability that could be considered as limitations of the procedure.

## Abbreviations

2-DE: Two dimensional electrophoresis
CgA: Chromogranin A
DRC: Dynamic range compression
MS: Mass spectrometry
PBA: Phenyl boronic acid
